# Plasma Concentrations of Extracellular DNA in Acute Kidney Injury

**DOI:** 10.3390/diagnostics10030152

**Published:** 2020-03-11

**Authors:** Jordanka Homolová, Ľubica Janovičová, Barbora Konečná, Barbora Vlková, Peter Celec, Ľubomíra Tóthová, Janka Bábíčková

**Affiliations:** 1 Institute of Molecular Biomedicine, Faculty of Medicine, Comenius University, 81108 Bratislava, Slovakia; homolova.jordanka@gmail.com (J.H.); lubica.janovicova@gmail.com (Ľ.J.); basa.konecna@gmail.com (B.K.); barboravlk@gmail.com (B.V.); petercelec@gmail.com (P.C.); tothova.lubomira@gmail.com (Ľ.T.); 2 Institute of Pathophysiology, Faculty of Medicine, Comenius University, 81108 Bratislava, Slovakia; 3 Department of Molecular Biology, Faculty of Natural Sciences, Comenius University, 84104 Bratislava, Slovakia; 4 Department of Clinical Medicine, University of Bergen, 5021 Bergen, Norway

**Keywords:** cell free DNA, uremia, ischemia, nephrectomy, ureteral obstruction

## Abstract

Current diagnostic methods of acute kidney injury (AKI) have limited sensitivity and specificity. Tissue injury has been linked to an increase in the concentrations of extracellular DNA (ecDNA) in plasma. A rapid turnover of ecDNA in the circulation makes it a potential marker with high sensitivity. This study aimed to analyze the concentration of ecDNA in plasma in animal models of AKI. Three different fractions of ecDNA were measured—total ecDNA was assessed fluorometrically, while nuclear ecDNA (ncDNA) and mitochondrial DNA (mtDNA) were analyzed using quantitative real-time PCR. AKI was induced using four different murine models of AKI-bilateral ureteral obstruction (BUO), glycerol-induced AKI (GLY), ischemia–reperfusion injury (IRI) and bilateral nephrectomy (BNx). Total ecDNA was significantly higher in BUO (*p* < 0.05) and GLY (*p* < 0.05) compared to the respective control groups. ncDNA was significantly higher in BUO (*p* < 0.05) compared to SHAM. No significant differences in the concentrations of mtDNA were found between the groups. The plasma concentrations of different fractions of ecDNA are dependent on the mechanism of induction of AKI and warrant further investigation as potential surrogate markers of AKI.

## 1. Introduction

Acute kidney injury (AKI) is a serious, often life-threatening condition with a multifactorial origin and increasing incidence [1,2]. The estimated incidence of AKI in hospitalized patients is one in five adults and one in three children and rising [2,3]. AKI is a common complication of major surgeries, traumas, and patients in the intensive care unit [4]. Surviving AKI is associated with declined quality of life, and it is currently considered the major risk factor for developing chronic kidney disease (CKD) [2]. Annual costs of management of AKI are estimated at USD 10 billion in the United States and USD 1.7 billion in the United Kingdom [5].

AKI is defined as an absolute increase in serum creatinine (sCr) by 0.3 mg/dL (26.5 µmol/L) within 48 h or by a 50% increase of sCr within seven days, or by a urine output less than 0.5 mL/kg/h for at least six hours [6]. The diagnosis of AKI is thus based on the measurements of serum creatinine, glomerular filtration rate, or oliguria [1]. Serum creatinine is routinely known as unreliable and significantly elevated only after severe damage to the kidneys [7]. Moreover, the sensitivity of serum creatinine to rapid changes in renal function is low, and it does not detect tubular injury [8]. Although significant improvements in investigating novel markers of AKI were recently reached, such as kidney injury molecule-1 (KIM-1), neutrophil gelatinase associated lipocalin-2 (NGAL) or Interleukin 18 (IL-18) (all mirroring injuries of proximal tubules), their incorporation into routine clinical practice is hindered by inconsistent results between different studies [2]. Thus, there is a need for biomarkers with the ability to detect kidney injury early, sustain high specificity, and rapidly monitor changes in renal function. 

Extracellular DNA (ecDNA) is a fraction of DNA which circulates in bodily fluids rather than being constrained inside the cell [9]. In plasma, it includes double-stranded (dsDNA) and single-stranded (ssDNA) DNA fragments of various length (tens to thousands of bp), mono or oligo-nucleosomes, extracellular traps (ETs), nucleolipidoproteic complexes, and microvesicular structures [10]. In healthy individuals, ecDNA originates predominantly from blood cells [11]. The turnover of extracellular DNA in the bloodstream is relatively fast, as demonstrated by the presence of placental ecDNA in women after delivery [12]. Following delivery, a high amount of ecDNA can be cleared from the maternal circulation within one hour, followed by additional slower phases within 2–13 h and complete removal of foreign ecDNA from the bloodstream within 48 h. The potential of ecDNA as a diagnostic tool has been widely explored during the last decades. In particular, the rapid clearance of ecDNA from the circulation is considered to be of potential interest for disease progression monitoring [13]. Although most of the studies focus on cancer, tissue damage in non-malignant conditions was found to be associated with high concentrations of ecDNA, namely stroke, myocardial infarction, and burns [14,15,16,17,18,19]. 

In CKD, the concentrations of ecDNA were not higher in patients than in healthy individuals [20,21]. On the contrary, in AKI, elevated concentrations of ecDNA were found in patients with sepsis who developed AKI compared to non-AKI sepsis [22]. Similarly, in animal models of AKI, elevated concentrations of ecDNA were found in ischemia–reperfusion injury [23,24] and glycerol-induced injury [25]. Based on the published results in the setting of acute kidney injury, we hypothesized that the concentrations of ecDNA following AKI might serve as a surrogate marker for the detection of AKI. This pilot study aimed to investigate the concentrations of different fractions of ecDNA using two different methods in four different animal models of AKI. 

## 2. Materials and Methods 

### 2.1. Animal Models of Acute Kidney Injury

Twelve week old C57Bl6 male mice were bred at the animal facility of the Institute of Molecular Biomedicine, housed 5 per cage, with ad libitum access to standard rodent chow and tap water, 12/12 light/dark cycle, constant temperature 22 ± 2 °C, and humidity 55 ± 10%. The ethical committee of the Institute of Pathophysiology approved all the experiments, number of approval 10/2015/SK1012 from 12 February 2015, name of the project: Extracellular DNA in Acute Kidney Injury.

All surgical procedures were performed under ketamine (100 mg/kg, Bioveta, Inc., Ivanovice na Hane, Czech Republic) and xylazine (10 mg/kg, Riemser Arzneimittel AG, Greifswall-Insel Riems, Germany) anaesthesia on a heating pad set to 37 °C. Wounds in all the procedures were sutured in two layers using a 5-0 silk. Bilateral ureteral obstruction (BUO) was performed by a midline incision and ligation of both ureters using 5-0 silk (*n* = 8 for BUO and *n* = 8 for SHAM) [26]. Ischemia–reperfusion injury (IRI) was performed in the left kidney by clamping both renal vessels using a vascular clamp (Fine Science Tools, Heidelberg, Germany). Ischemia was maintained for 30 min, the clamp was removed and the reperfusion was confirmed macroscopically. The contralateral kidney was removed afterwards. Unilateral nephrectomy (UNx) was carried out by removing the right kidney (*n* = 8 for IRI, *n* = 5 for UNx and *n* = 3 for SHAM) [27]. Bilateral nephrectomy (BNx) was performed by midline incision followed by decapsulation of both kidneys and ligation of renal vessels and ureters using 5-0 silk followed by excision of both kidneys (*n* = 4 for BNx and *n* = 4 for SHAM) [28]. SHAM operated animals underwent the same procedures without the ligation, excision, or clamping. Glycerol-induced AKI (GLY) was induced by intramuscular hind limbs injection of 50% glycerol (8 mg/kg). Control mice (CTRL) were injected with physiological saline [29]. Mice were sacrificed either 48 (BUO, GLY) or 24 h (IRI, BNx) after induction of the injury. 

### 2.2. Blood Collection and Tissue Processing for Light Microscopy and Histological Analysis

Blood was collected via retro-orbital puncture using EDTA tubes (Sarsted, Nümbrecht, Germany), centrifuged at 1600 g and stored at −20 °C until analysed. The kidneys were removed, cut longitudinally, fixed in Methyl Carnoy’s solution overnight, dehydrated, and embedded in paraffin. Sections (1 μm) were stained with Periodic Acid Schiff (PAS) stain according to the standard protocols. Tissue injury, with focus on tubular dilation, atrophy, and the presence of proteinuric casts, was analysed to confirm the renal injury. 

### 2.3. Biochemical Analysis

Plasma creatinine and blood urea nitrogen (BUN) were analysed using colorimetric detection kits according to the protocol provided by the manufacturer (Arbor assays, Ann Arbor, Michigan, USA). 

### 2.4. Analysis of EcDNA

EcDNA was isolated from 200 μL of plasma using the QIAmp Blood Mini Kit (Qiagen, Hilden, Germany) according to the manufacturer’s instructions. 

For total DNA, the Qubit Fluorometer and Qubit dsDNA HS Assay Kit (Thermo Fisher Scientific, Waltham, Mass) was used according to the instructions by the manufacturer. ncDNA and mtDNA were analysed using real-time PCR Mastercycler realplex 4 (Eppendorf, Hamburg, Germany). The PCR was performed using the QuantiFast SYBRGreen PCR Kit (Qiagen, Hilden, Germany), designed for the absolute quantification of low-copy DNA targets. The primers and DNA template were added according to the instructions. The following program was used: 1 × 5 min at 95 °C; 40 × 10 s at 95 °C for denaturation, 30 s at 60 °C for annealing and 30 s at 75 °C for extension. For mitochondrial DNA, the following primers for cytochrome c gene were used: F: 5’-CCCAGCTACTACCATCATTCAAGT-3’; R: 5’-GATGGTTTGGGAGATTGGTTGATGT-3’ [30]. For nuclear DNA, primers for beta-2-microglobulin gene were used: F: 5’-TGTCAGATATGTCCTTCAGCAAGG-3’; R: 5’-TGCTTAA CTCTGCAGGCGTATG-3’ [31]. The relative units, shown as percentages, were calculated from the absolute cycle threshold (CT) values using the standard curve designed as serial dilutions of purified PCR products of known independent samples for each target gene.

### 2.5. Statistical Analysis 

Data were analysed using IBM SPSS Statistics Version 25.0 (New York, NY, USA). One-way analysis of variance (ANOVA) followed by Bonferroni’s post hoc test were used to analyse the results. The *p* values less than 0.05 were considered statistically significant. All data are presented as mean + SD. 

### 2.6. Data Availability Statement

The Excel data used to support the findings of this study are available from the corresponding author upon request.

## 3. Results

### 3.1. Biochemical and Histological Analyses Confirm AKI

**Figure 1:**
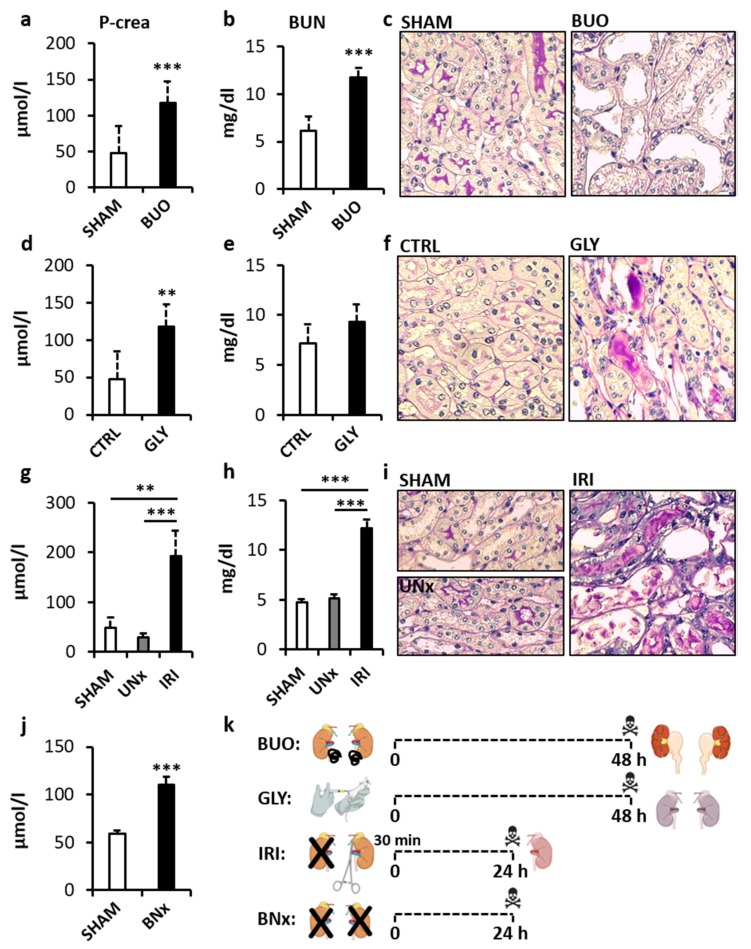
Renal function and histopathological assessment of mice with acute kidney injury (AKI). Bilateral ureteral obstruction: (**a**) creatinine; (**b**) BUN; (**c**) left panel—normal renal architecture, right panel—tubular dilation and atrophy. Glycerol-induced AKI: (**d**) creatinine; (**e**) BUN; (**f**) left panel—normal renal architecture, right panel—tubular atrophy and proteinuric casts. Ischemia–reperfusion injury: (**g**) creatinine; (**h**) BUN; (**i**) left panel—normal renal architecture, right panel—tubular atrophy and dilation, epithelial cell necrosis and cell shedding, and proteinuric casts. Bilateral nephrectomy: (**j**) creatinine. Overview of the preparation of the individual models (**k**). BUO—bilateral ureteral ligation, BUN—blood urea nitrogen, GLY—glycerol-induced AKI, UNx—unilateral nephrectomy, IRI—ischemia–reperfusion injury, BNx—bilateral nephrectomy. ** denotes *p* < 0.01, *** denotes *p* < 0.001. Data are presented as mean + SD. Histological images show periodic acid Schiff stain (PAS), magnification 400x.

In the first step, successful induction of AKI in all animal models was confirmed. The bilateral ureteral obstruction (BUO) model showed +101% and +92% higher concentration of plasma creatinine and blood urea nitrogen (BUN) compared to SHAM-operated animals, respectively (Figure 1a,b, *p* < 0.001, both; *t* = 5.63 and *t* = 3.93, respectively). In the glycerol-induced AKI (GLY), plasma creatinine was higher by +146% and BUN by +30% compared to CTRL group (Figure 1d,e, *p* < 0.05 and *p* = 0.16; *t* = 4.53 and *t* = 1.62, respectively). Both creatinine and BUN were significantly different in the ischemia–reperfusion injury (IRI) model (*p* < 0.001 both; *F* = 26.73 and *F* = 142.26, respectively). Creatinine was higher by +297% compared to SHAM and by +556% compared to UNx group (*p* < 0.01 and *p* < 0.001, respectively, Figure 1g); BUN was higher by +156% compared to SHAM and by +138% compared to UNx (*p* < 0.001, both, Figure 1h). The bilateral nephrectomy (BNx) model showed a higher concentration of plasma creatinine by +86% compared to SHAM operated controls (Figure 1j, *p* < 0.001; *t* = 10.84). A histological examination of kidneys from SHAM operated, vehicle-treated, or uninephrectomized animals showed normal renal histology (Figure 1c,f,i; left panels). In BUO, a histopathological evaluation of PAS stained kidney sections showed marked dilation and atrophy of tubular segments (Figure 1c; right panel). The GLY group showed proteinuric casts and tubular atrophy (Figure 1f; right panel). The IRI group showed tubular atrophy and epithelial cell necrosis and cell shedding, tubular dilation, and multiple proteinuric casts (Figure 1i; right panel).

### 3.2. Total EcDNA is Elevated in Animal Models of AKI

**Figure 2:**
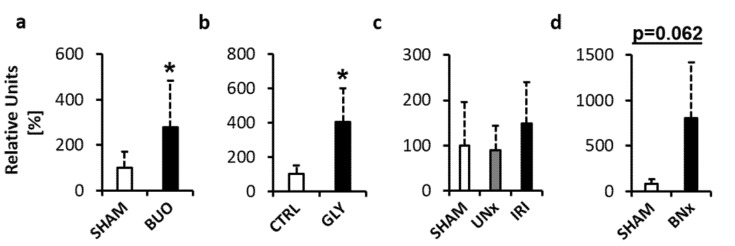
Total ecDNA measured in plasma of mice with acute kidney injury (AKI). (**a**) Bilateral ureteral obstruction. (**b**) Glycerol-induced AKI. (**c**) Ischemia–reperfusion injury. (**d**) Bilateral nephrectomy. * denotes *p* < 0.05. Data are presented as mean + SD. BUO—bilateral ureteral ligation, GLY—glycerol-induced AKI, UNx—unilateral nephrectomy, IRI—ischemia–reperfusion injury, BNx—bilateral nephrectomy.

The first Fluorescent assessment of extracellular DNA in plasma provides the measurement of total DNA in plasma, regardless of its origin. Total ecDNA was higher by +177% in BUO compared to the SHAM operated group (Figure 2a, *p* < 0.05; *t* = 2.31). In GLY, total ecDNA was higher by +304% compared to the CTRL group (Figure 2b, *p* < 0.05; *t* = 2.99). In IRI, there were no significant differences between the groups (*p* = 0.41; *F* = 0.95). Total ecDNA was higher by +49% and by +68% when compared to SHAM and UNx (Figure 2c, *p* = 0.659 and *p* = 0.428, respectively). In BNx, total ecDNA was higher by +891%, but the difference was not statistically significant (Figure 2d, *p* = 0.062; *t* = 2.90). 

### 3.3. Nuclear EcDNA is Elevated in the BUO Model 

**Figure 3:**
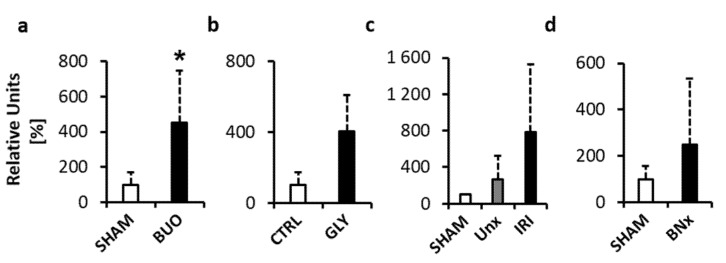
Nuclear ecDNA (ncDNA) measured in plasma of mice with acute kidney injury (AKI). (**a**) Bilateral ureteral obstruction. (**b**) Glycerol-induced AKI. (**c**) Ischemia–reperfusion injury. (**d**) Bilateral nephrectomy. * denotes *p* < 0.05. Data are presented as mean + SD. BUO—bilateral ureteral ligation, GLY—glycerol-induced AKI, UNx—unilateral nephrectomy, IRI—ischemia–reperfusion injury, BNx—bilateral nephrectomy.

To analyse whether the ecDNA in plasma originates from the nucleus (ncDNA), real-time RT PCR targeting a chromosomal gene (beta-2-microglobulin) was performed. In the BUO model, ncDNA was significantly higher in the experimental group by +353% when compared to SHAM (Figure 3a, *p* < 0.05; *t* = 3.32). The GLY group had a higher concentration of ncDNA by +396% when compared to the CTRL group, but the difference was not statistically significant (Figure 3b, *p* = 0.125; *t* = 2.07). In the IRI model, there were no significant differences between the groups (*p* = 0.17; *F* = 2.05). Total ecDNA was higher by +681% when compared to SHAM and by +160% when compared to UNx (Figure 3c, *p* = 0.214 and *p* = 0.326, respectively). In the BNx model, the ecDNA concentration was higher by +148%, but the difference was not statistically significant (Figure 3d, *p* = 0.348; *t* = 1.02). 

### 3.4. Mitochondrial EcDNA is not Elevated in Animal Models of AKI

**Figure 4:**
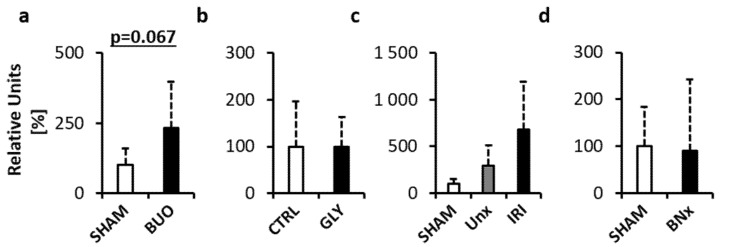
Mitochondrial ecDNA (mtDNA) measured in plasma of mice with acute kidney injury (AKI). (**a**) Bilateral ureteral obstruction. (**b**) Glycerol-induced AKI. (**c**) Ischemia–reperfusion injury. (**d**) Bilateral nephrectomy. Data are presented as mean + SD. BUO—bilateral ureteral ligation, GLY—glycerol-induced AKI, UNx—unilateral nephrectomy, IRI—ischemia–reperfusion injury, BNx—bilateral nephrectomy.

To analyse the involvement of mitochondrial DNA (mtDNA), real-time RT PCR targeting a mitochondrial gene (cytochrome c) was performed. The analysis showed a higher concentration of mtDNA in BUO compared to the SHAM group by +132%, but the differences did not reach statistical significance (Figure 4a, *p* = 0.067; *t* = 2.09). In the GLY model, no differences were found between the groups (Figure 4b, *p* = 0.985; *t* = 0.02). There were no significant differences between the groups in the IRI model (*p* = 0.11; *F* = 2.66). IRI had a higher concentration of mtDNA by +850% compared to SHAM and by +133% compared to UNx (Figure 4c, *p* = 0.133 and *p* = 0.303, respectively). There were no differences observed between the groups in the BNx model (Figure 4d, *p* = 0.911; *t* = 0.12).

## 4. Discussion

The present study analyzed the concentrations of ecDNA in plasma of three different animal models of AKI and one model of uremia. Three different fractions of ecDNA were analysed. The fluorometric analysis was performed to analyze the unspecific ‘total’ ecDNA, while real-time (RT) PCR was used to detect DNA of nuclear and mitochondrial origin. This study shows that there are differences in the concentrations of different fractions of ecDNA, likely depending on the mechanisms of induction of renal disease and uremia.

Under physiological conditions, most of the circulating ecDNA molecules are of hematopoietic origin [32]. After tissue injury, the equilibrium shifts, and the main source of ecDNA is primarily the affected organ, as described for acute myocardial infarction, cancer, or solid organ xenografts [18,33,34]. In addition, as a response to injury, leukocytes may undergo a process of ETosis and form extracellular traps (ETs) with antimicrobial and immune activity [35]. So far, neutrophils (neutrophil extracellular traps, NETs), macrophages (macrophage extracellular traps, METs), eosinophils, basophils, and mast cells were found to be able to undergo ETosis [36,37,38,39,40]. The formation of ETs includes the disruption of the nuclear membrane, chromatin decondensation, and mixing with the context of cytosol and final extrusion of a net-like structure composed of DNA, histones, proteases, and antimicrobial peptides into the extracellular space [41]. Although the DNA in the ETs was first described to be of nuclear origin, recent findings have shown that under certain conditions, ETs formed by eosinophils can contain both ncDNA and mtDNA and neutrophils can form NETs containing only mtDNA [38,42,43]. 

The three different models of AKI used in the present study all originated from a different type of injuries to the kidneys. Still, on the cellular level, the primary target cells in all of the models are within the tubular segments of the nephron. In BUO, the injury is caused by the increased strain on the nephrons, which results in tubular dilation and atrophy, followed by an early infiltration by inflammatory cells, mostly macrophages and lymphocytes [44]. Thus, the most likely source of the elevated ecDNA levels in this model are the tubular cells and possibly early inflammatory infiltrates that underwent ETosis, but the latter has not yet been described in this model. 

Due to the high abundance of mitochondria, the metabolically highly active proximal tubular cells are the primary target of injury in acute IRI. Markedly, the S3 segment of the proximal tubule in the corticomedullary region is the most affected area due to the high oxygen demands compensating for the physiologically hypoxic medullary region [45]. Based on this, it was expected that the mtDNA would be highly increased in this model. Nevertheless, even though the mtDNA in this model was higher by 850% compared to SHAM animals, the results were not significantly different. On the other hand, compared to the BUO model, where both proximal and distal tubular cells are affected more equivocally, the average fold increase in mtDNA levels in BUO (+133%) was lower when compared to the IRI model (+850%). Unfortunately, due to the high inter-individual variability between the mice in this preliminary experiment, it can only be speculated that the addition of more animals per group would increase the statistical power of the analysis and confirm the assumption. Experimental data on the plasma mtDNA levels in AKI are sparse, and so far has only been described in septic AKI, where the elevation in plasma mtDNA appeared due to the sepsis [46]. Most of the researchers focused their research on the urinary levels of mtDNA. In mice after IRI, urinary mtDNA was elevated significantly 24 h after 10 or 15 min of ischemia [47]. The same study also showed higher urinary mtDNA in patients with progressive AKI after cardiopulmonary bypass. Similarly, patients with hypertensive nephropathy showed higher levels of mtDNA in urine compared to healthy volunteers [48]. It should be noted that a high level of fragmentation of mtDNA in plasma might hinder its analysis using real-time PCR, likely making urine a superior biofluid for these analyses [49].

Although both total ecDNA and ncDNA showed an increasing trend in our experiment, the results did not reach statistical significance, probably due to high inter-individual variability in our samples. Elevated levels of plasma ecDNA in IRI is in line with other studies and the discrepancies in the significance of the results might be due to a different fluorescent method used and the use of real-time PCR in our study [24].

The pathophysiology of the GLY model includes rhabdomyolysis, followed by myoglobin-induced nephrotoxicity in the kidneys [50,51]. The key mechanisms, although not fully elucidated, include oxidative stress, endothelial dysfunction, inflammation, and apoptosis. Histologically, the rhabdomyolysis-induced AKI is manifested mainly by cortical tubular cell necrosis and inflammatory cell infiltration. Our results in this model showed significantly increased total ecDNA with a similar pattern (although not statistically significant) of the ncDNA. Interestingly, there was no trend in the increase of mtDNA levels when compared to the control group in this model. The reason for this might be the different pathophysiology of injury to the kidneys, which, compared to the previous two models (BUO, IRI) originates from a systemic injury to skeletal muscles rather than in the kidneys per se. Recent studies showed that the infiltrating macrophages via the formation of METs are crucial to promoting injury to the kidneys in the model [25,52]. Moreover, in our experiment, the overall histologically detectable injury in the GLY model was milder compared to the BUO and IRI models (Figure 1), also reflected in only a mild elevation of the levels of BUN, suggesting mtDNA as a potentially more suitable marker of intrinsic renal AKI rather than systemically induced AKI.

The BNx model represents a model of severe uremia, which allows the study of the systemic effects of the uremic toxins without any residual filtration capacity of the injured kidneys [53]. The levels of total ecDNA were marginally significant (*p* = 0.062), and the ncDNA showed an increase by +148% on average. The observed trend of the increase is likely the result of a peripheral tissue injury caused by uremic toxins. The state of uremia leads to a systemic inflammation with the migration of inflammatory cells, altered expression of both pro and anti-inflammatory cytokines, disruption of homeostasis, and the oxidation-reduction status of the organism [54]. Distant organ dysfunction as a complication of AKI and uremia was reported in the lungs, brain, heart, liver, and the gastrointestinal tract [55,56,57]. Thus, the most probable source of ecDNA in this model is the injured cells in the peripheral tissues and immune cells undergoing ETosis. Interestingly, because the levels of mtDNA were not elevated in this model, it supports the assumption that the injured tubular cells might be the primary source of mtDNA in the BUO and IRI models.

Another aspect that should be taken into consideration is the clearance of ecDNA, since there is no residual renal filtration in this model. Although the mechanism by which ecDNA is cleared from the circulation is not entirely known, the liver was found to have an important role in the clearance of single-stranded DNA (ssDNA) and mononucleosomes in mice [58,59]. The kidney, despite being the primary excretory organ, seems to play only a minor role in the removal of ecDNA from plasma, as was demonstrated for both native and denaturated DNA injected into the mice [60]. In addition, haemodialysis increases ecDNA for a limited time (probably due to the mechanic stress on the circulating blood cells), but the longer-term effect of blood filtration does not influence the levels of ecDNA in CKD patients before the next dialysis session, and these levels are comparable to healthy individuals [20]. 

On the other hand, a different mechanism of clearance by renal filtration was found in the urine of pregnant women, with differences between maternal and fetal ecDNA [12]. Interestingly, the time-lapsed clearance profile of maternal ecDNA in the urine was dependent on the size of the fragments, while no such phenomenon was observed for fetal ecDNA. Nevertheless, renal filtration is not the only mechanism involved in the elimination of ecDNA. The most abundant endonuclease in the kidney is Deoxyribonuclease I (DNAse I) [61]. The expression of the renal DNAse I in the injured kidney seems to be dependent on the mechanism of injury. While lupus nephritis was associated with decreased expression of the enzyme in both animal models and humans, cisplatin induced injury and IRI were shown to increase the expression of the enzyme [61,62,63]. 

Taken together, the renal filtration and renal endonucleases are partially responsible for the eradication of ecDNA from circulation, and their function is altered in renal injury. However, the renal route is only responsible for less than 20% of the total ecDNA that is being cleared from the circulation by kidneys [12]. It is thus likely that the uremic environment, rather than the absence of the clearance capacity of the kidneys, is responsible for the elevated levels of ecDNA in this model. 

This study comes with several limitations. The limiting factors were plasma volume, high inter-individual variability, and the inability to analyze urine samples due to the low volume of collected urine. In addition, due to the ethical reasons, it was impossible to perform repeated blood collection from the mice, and the study of the measurement of the concentration of ecDNA was limited to the time at sacrifice. Repeated blood collections at earlier stages after the injury might give a better overview of the dynamics in ecDNA upon kidney injury. Because this was a pilot study to analyze whether the levels of ecDNA are elevated in AKI irrespective of its origin, the cellular sources of ecDNA were not assessed. Moreover, our currently unpublished data suggest that the centrifugation step, namely re-centrifugation of the collected plasma at a higher speed, improves the informative value of ecDNA analysis. Detailed correlation of the different fractions of ecDNA with standard parameters of renal function will be the focus of the following studies.

In conclusion, the present study demonstrated that the concentrations of ecDNA in plasma in AKI are dependent on the mechanism of induction of AKI. Moreover, different fractions of ecDNA showed a different pattern of elevation based on the animal model used. Further studies warrant closer analysis of the ecDNA found in plasma in AKI.
